# Transactional Sex among Youths in Post-conflict Liberia

**DOI:** 10.3329/jhpn.v29i2.7853

**Published:** 2011-04

**Authors:** Katharine A. Atwood, Stephen B. Kennedy, Ernlee M. Barbu, Wede Nagbe, Wede Seekey, Prince Sirleaf, Oretha Perry, Roland B. Martin, Fred Sosu

**Affiliations:** ^1^Pacific Institute for Research and Evaluation, Louisville Center, 1300 S Fourth Street, Suite 300, Louisville, KY 40208, USA; ^2^UL-PIRE Africa Center, HIV/STD Prevention Research Center, A.M. Dogliotti College of Medicine, University of Liberia, Monrovia, Liberia

**Keywords:** Acquired immunodeficiency syndrome, HIV, Sex behaviour, Transactional sex, Youth, Liberia

## Abstract

This paper presents findings on sexual risk behaviours of Liberian youths based on five focus-group discussions conducted with 6th and 7th graders (n=36) attending an elementary/middle school in Monrovia, Liberia. The purpose of the focus-group discussions was to gain an understanding of the sexual behaviours of in-school Liberian adolescents. The focus-group discussions were part of a larger study to adapt an evi-dence-based HIV-prevention intervention—Making Proud Choices!—for in-school youths. Post-conflict conditions were discussed as a contributor to the emergence of high-risk sexual behaviours, including transactional sex, sexual violence, and lack of condom-use. Transactional sex was often described by the focus-group participants as occurring between young females and older, more financially-secure males to obtain cash, food, clothing, western commodities, and school-fees and was often encouraged by parents and promoted by peers. The findings also indicate that female adolescents make choices to engage in transactional sex to gain access to a continuum of material and consumer needs. These findings suggest that individual risk-taking behaviours are nested within complex sexual economies and that HIV-prevention interventions should be considered that leverage females’ agency and control.

## INTRODUCTION

The two-decade civil war in Liberia resulted in 270,000 casualties. Thousands more were tortured, maimed, or victimized by sexual violence, including being held as sexual slaves or exchanging sex for protection, shelter, and food. During the civil conflict, the domestic gross national product (GNP) in Liberia fell 90% from 1987 to 1996, one of the largest ever-recorded recessions (1). The civil conflict ended eight years ago (2003), and in 2005, Liberia held its first democratic election and is now witnessing a remarkable re-investment by the international donor communities in all sectors of its economy (1, 2). Despite these efforts, the economy of Liberia is plagued by 85% unemployment (3-4), insufficient domestic and human capital (2, 4), and a destroyed physical and economic infrastructure (4).

Little is known about the rates of HIV prevalence in Liberia, and detection efforts are hampered by a severely-damaged public-health system and loss of trained personnel (4-6). The most recent Liberian Demographic Health Survey indicates that 1.5% of people aged 15-49 years are HIV-infected. The prevalence rates are higher among females than among males (1.8% vs 1.2%), indicating a low-level gene-ralized epidemic (7). Gender differences in prevalence rates are greater for those aged 15-25 years, suggesting that young females are at a greater risk of infection than males of the same age-group (7). In Monrovia, the nation's capital, the rate of HIV infection is 2.8% for women and 2.1% for men. Surveillance studies of women attending antenatal clinics in 10 urban centres found an overall prevalence rate of 5.4% (8).

The Ministry of Health and Social Welfare of Liberia reports that the primary focus of heterosexual transmission is among older males and younger females (9). It is suggested that transactional sex emerged in Liberia during the conflict and post-conflict periods as a means of coping with scarce resources and limited economic opportunities (4), although few published qualitative or quantitative studies in Liberia have examined this issue to date (10).

Results of studies in sub-Saharan Africa indicate that transactional sex tends to occur in exchange for cash, goods, and services (11-13), school-fees (12, 13), advancement (11, 13, 14) or to gain heightened social status, and it is cited as placing women at an increased risk of HIV infection (14-17). These transactions often occur between older male adults and young females where power differentials—the inability to negotiate condom-use and coercion—contribute to women's risk of HIV/sexually transmitted diseases (STDs), unwanted pregnancy, and sexual violence (17-20). Transactional sex has also been suggested to be part of a larger pattern of social interdependence in sub-Saharan Africa where women receive material goods while males gain outward displays of power and prestige (11, 21). With the uncertainty that surrounds daily life in these countries, women may develop several concurrent transactional sex partners as a form of ‘social insurance’ (21) providing them with resources to call upon, should crises such as famine or war re-emerge (21, 22). For the purposes of this study, transactional sex was defined as engaging in sexual intercourse in exchange for cash, goods, services, commodities, or privileges that are perceived as needs or wants by the participant.

While it is well-established that transactional sex places women at a risk of sexual violence, unprotected sex, early pregnancy, and HIV infection (15, 23-25), several researchers have critiqued the analysis of women as primarily victims of transactional sex and men as the perpetrators (26-29). These researchers call for a broader understanding of transactional sex as nested in complex sexual economies where women make choices to engage in transactional sex to meet a broad array of needs in resource-constrained communities (26-28).

Similar to other post-conflict countries, Liberia has witnessed the establishment of a large and successful peace-keeping infrastructure to restore peace and rebuild the governmental infrastructure (1). Local entertainment industries have been established and created what Jennings refers to as ‘peacekeeping economies’ (10). Peacekeeping economies may provide a relatively-affluent pool of potential transactional sex partners to engage in sex in return for western commodities, goods, services, and possible employment (10). While the United Nations (UN) has established a ‘zero tolerance’ policy and sanctions for the engagement in transactional sex among UN personnel (30), such sanctions can be difficult to enforce and are said to potentially drive the behaviour underground (10).

There are no recent quantitative or qualitative publications about transactional sex or other high-risk sexual behaviours of Liberian youths or how post-conflict conditions may contribute to these behaviours. This paper helps fill these deficits in the literature by providing the first known qualitative findings about the sexual behaviours of in-school youths as part of a larger study to provide an evidence-based HIV-prevention intervention to Liberian youths. The objectives of the qualitative study were to understand: (a) circumstances and motivators for Liberian adolescents to get involved in sex, (b) pressures to engage in sex, and (c) perceptions and attitudes of adolescents towards condom-use. We present these findings and discuss their implications for HIV-prevention interventions targeting Liberian adolescents.

## MATERIALS AND METHODS

### Eligibility criteria

Participants were eligible to participate in the study if they were aged 13-19 years and were enrolled in the 6th or 7th grade of a public elementary/middle school in Monrovia, Liberia. The age range was broad because in-school youths are slightly older having returned to school only after increasing stability and security in 2005 (2). All the participants came from one public school. The school is set within a 10-mile section of the capital—Monrovia—a city with a population of 1.2 million (31).

Members of the Liberian research team met the 6th and 7th grade students at school and described the objectives, methodology, risks, and benefits and asked for male and female participants. A convenience sample of 36 students was invited to participate in the focus-group discussions. All agreed to participate. Focus groups were selected, as opposed to one-on-one interviews, to allow for a more open exchange of ideas. In addition, our Liberian colleagues expressed concern that adolescents may be hesitant to talk privately about sensitive issues with an adult interviewer. Five focus-group discussions were conducted. All discussions were in English, the national language in Liberia.

### Focus-group study participants

Students (n=36) were stratified by gender and randomly assigned to one of four same-gender focus groups and one mixed gender focus group. Their age ranged from 13 to 19 years (mean=16.5 years), and 50% were male. The mixed gender focus group, although not typically used for eliciting sensitive information, allowed us to examine attitudes that might emerge in a typical classroom, mirroring the setting for our adapted HIV-prevention intervention.

### Focus-group administration procedures

Two focus-group moderators were assigned to each group according to gender. The mixed gender focus group was moderated by a male/female focus-group moderator team. Before the start of the focus-group discussion, the consent forms were reviewed, eligibility re-confirmed, and confidentiality stressed. Students were instructed that they would discuss perceived risk behaviours of students of their age. Each focus-group discussion lasted for 60-90 minutes.

### Analysis of data

Focus-group discussions were audio-taped and transcribed. Transcripts were compared with the tapes to check for accuracy. Transcripts were analyzed within the conceptual framework of qualitative research, using a coding and thematic analysis approach (32-36). Two Liberian/US review teams reviewed all transcripts to elucidate key quotes within the context of the Liberian culture. Codes were placed alongside key quotes by each review team. Codes were shared to arrive at a mutually-agreed upon coding scheme. Major themes were identified based on these codes. Themes were prioritized according to the views expressed by the majority of the participants.

Results are presented by thematic topic. The dominant theme that emerged related to transactional sex, including peer and parental pressures, to engage in transactional sex. Other themes included barriers to condom-use, sexual violence, and identification of high-risk settings where sexual risk behaviours occur.

### Ethics

The Institutional Review Boards of the Pacific Institute for Research and Evaluation (PIRE) in the USA and the University of Liberia approved the human subject protocols in accordance with the Helsinki Declaration (37). Interested students received a parental consent form. The consent form indicated that participation was voluntary, and the focus groups would ask about sexual risk behaviours of students of their age and that the information would be audio-taped, remain confidential, and would not be shared with school administrators, teachers, or linked to individual students. Students received refreshments, notebooks, and pencils as incentives for participation. All audio-tapings were destroyed after typed transcriptions were completed by the Liberian research team members. Research materials were placed in locked cabinets at the PIRE research office with access limited to the research team members. No names were associated with any of the focus-group participants.

Members of our Liberian research team attended a three-day training on focus-group methodologies. Three mock focus groups were conducted with 6th and 7th grade students (n=24) from a neighbouring elementary school to provide practice for moderators. Emphasis was placed on encouraging participants to reflect on experiences of in-school youths of similar age but not to divulge their own personal stories. To promote greater rapport, all focus-group moderators were Liberians aged 23-25 years. All the moderators had previous experience in conducting adolescent focus-group discussions for an ongoing NIH-funded community-based HIV-prevention study.

## RESULTS

### Transactional sex—provides financial freedoms and respect

When the participants were asked about their motivation for becoming sexually-active, there was a general recognition among girls that their sexuali-ty had value on the sexual exchange market and provided certain financial freedoms, status, and power. Although many young women reported that first sexual encounters were of a non-transactional nature, they described becoming involved in transactional sex after witnessing the financial gains and heightened social status of peers. Both female and male respondents frequently referred to transactional sexual exchanges as ‘Man-Man’ business, and transactional sex partners were referred to as ‘Big, Big Men’. ‘Big, Big Men’ is a Liberian expression used to describe men of affluence, power, and status in Liberian communities (38), suggesting substantial age differences between the female adolescents and their transactional sex partners.

Transactional sex appeared to provide adolescent females with a type of social agency, within the confines of their difficult economic circumstances, which enabled them to participate in the post-conflict economy without feeling left behind.

One female respondent reported:

Those doing the Man-Man business are more popular. Those who are not having sex cannot go anywhere. They just sit at home.

Another female respondent commented:

Because they go out with Big-Big men, we can respect them.

One female respondent stated:

If you see your friends with so much money, you ask her to assist you with US$10, they will say, “Go and do your own Man-Man business, and you will have your own money”…. Since she is in it, I will join her too. That is just how we get involved in sex.

Male adolescents reported pressure to become involved in transactional sex. The male adolescents seemed to be shaped by a hegemonic masculine identity of power defined by affluence and promiscuity. One male respondent reported:

The main thing in getting us involved in sex is the world we find ourselves in today, people seeing television, we are carried away by persons having money, you see the man passing with girlfriends and things and you say ‘as long as this man can get a girlfriend, I myself can get a girlfriend.’

Male adolescents viewed money as their access point, “if you have money, you can get involved in women business”, reinforcing the commoditization of sex among Liberian youths.

### Transactional sex—competing parental expectations

Focus-group participants described parental pressure to become involved in sex to obtain needed commodities for the household.

A female respondent reported:

Some mothers encourage us to go on the street and join it. Even if you do not want to, they will encourage you to do it. They will say, “you are big enough so you should not go to school, just join it.”

One young male commented on how this develops within families:

If your background is poor and your sister is the only girl in the family, they (family) see this Big Man coming to say “I want your daughter.” The family will force her saying “you know we do not have anything. At least when you love this man—the man will do things for us.”

Although the adolescents reported parental pressure to engage in transactional sex, there was an implicit recognition of conflicting parental expectations. Parents wanted involvement of their daughters to remain discrete so “our futures will not be damaged.” These findings are similar to those of other studies of parental attitudes towards transactional sex conducted in Tanzania (26, 39) where parents wanted to preserve a public perception that their daughters were not sexually active to strengthen marriage prospects. The adolescent males shared similar attitudes, reporting that females may become ‘damaged before their time’ and ‘have no future’. Males did not ascribe any concerns or perceived risks about their own involvement in transactional sex.

### Transactional sex—limits the ability to refuse sex or negotiate condom-use

In addition to helping families with material goods, transactional sex partners were reported to assist with school-fees while teachers sometimes elicit sex in exchange for better grades. These interactions and inherent power differentials were reported to make it difficult to refuse sexual advances or negotiate condom-use. Sex tended to be viewed as a commodity and once purchased, the male partner maintained power over the sexual encounter.

One female focus-group participant reported:

Some girls cannot read, so when the man says, “come sleep with me”, then they will go to sleep with him for a grade.

One female respondent commented:

… They will want to have you any way they want…. Since they are giving you money, you would not have any choice to say, “I do not want to have sex without a condom.”

The involvement of male adolescents in transactional sex carried with it similar assumptions about male dominance and sexual control. One respondent commented:

If a boy wants to go three or four rounds, she can say, “you are going to kill me” but the boy can say, “I pay my money to enjoy good things.”

### Transactional sex—impact on partnership selection

Although male respondents described being involved in transactional sex, female respondents reported that they had sex with males of similar age the first time “because we would be afraid with someone older.” As their sexual involvement grew, they became aware of their worth on the sexual exchange market, and partnerships with more affluent males were found to reap greater rewards.

A female focus-group participant reported:

You have an older man and you have a young boy of your age. You will say, “What does this young boy have to offer me?” The older man will give me something to buy things for myself.

Another respondent commented:

The equal friends (boys of the same age) do not have money to give them; so, they will look for someone who has it. So, they start loving their “Pa Crowd” or their “Uncle Crowd”.

### Perceptions of transactional sex—emerging from the war but parents blamed for its continuation

The participants consistently commented that transactional sex began “because of the war” or “since the war began”. While most respondents spent the majority of their lives under civil and post-conflict circumstances, they suggested that sexual norms were different before the war.

While participants acknowledged that transactional sex remained a common practice after seven years of political stability, they tended to blame parents for allowing the practice to continue and for its emotional toll on young girls.

One female respondent said:

There is a danger in there. The reason why it is not good to love an older man is because that person and your father are equal. How will you feel? You will not feel fine.

One male respondent commented:

It makes you lose respect for older people; you start to disrespect your parents at home.

### Venues for sexual risk-taking among Liberian youths—video clubs

Video clubs in Liberia have been described as small enterprises, allowing consumers to view sexually-explicit media while providing a private venue where sex can be exchanged for entry and where alcohol is available (38). In focus groups, female respondents reported that one can gain entry from ‘bad boys’ and “follow them into rooms for small money”. They portrayed out-of-school girls as more likely to engage in sexual risk behaviours because they can “spend the whole day in the video club” and follow their “video club boyfriends to their rooms”. Respondents indicated that one had a degree of control when navigating video clubs: “Myself I can go to the video club but it is left up to you, whether you want to entertain yourself or look for a video club boyfriend.”

### Perception of sexual violence and rape

As noted previously, sexual violence and gang rape were widespread during the two decades of civil conflict (40) and have continued during the post-conflict period (41). In our focus groups, female respondents indicated that rape can occur during transactional sex if they refuse sex or insist on condom-use, highlighting the expression of male power through sexual violence.

One respondent commented:

Big-Big guys may invite a girl to his house …. Maybe he brings up sex, and if the girl says ‘no’, he will end up forcing her when nobody is around.

Sexual violence was also said to occur between females and males of similar age. Both sexual and criminal violence were communicated as significant issues in their villages.

One female respondent commented:

They come outside with armed robbers in the night, and they put the armed robbers behind the house. They can be crying “my people come help me”**, …** when you get outside, they jump on you and hurt and rape you.

Male focus-group participants assigned some level of blame to the victim and pointed to crowded living conditions as a contributor to sexual violence. One male respondent reported:

One girl in my area, she wears short clothes. Our hearts are quick to cut that way. A guy who lives in the same house with her and her brother saw her lying down with her trouser high, when he saw it, he jumped on her and raped her.

### Attitudes towards condom

Participants viewed condoms as a means of keeping them in school by preventing pregnancy and early marriage. One respondent commented:

If we use condoms, we do not get pregnant; so, we can go to school and prepare our future. Another person commented: if you use condoms, it will help you reach your goals, like staying in school.

While there was a general recognition that condoms prevent pregnancy, female respondents reported that condoms can “damage the womb” or cause “pain in the belly”, and when using condoms, “boys can be too rough”. The female respondents seemed to conflate “rough sex” with the possibility of damaging their reproductive organs, highlighting the importance of fertility for female identity and power. They indicated that adolescent males and Big Men liked it “flesh to flesh” or “blood to blood”.

In contrast, male respondents felt that if they paid for sex, they did not have to use a condom. One male respondent commented: “When you put your money up, you must get the good thing.” They said that their female partners would be insulted if they insisted on condom-use: “She will say that it is a street-girl who uses a condom.” To convince girls to have unprotected sex at sexual debut, one male respondent said: “we tell them that the first time cannot make them pregnant.” Males did not express any perceived risks for not using a condom.

Only when prompted, did the participants discuss fears of AIDS from unprotected sex. Female res-pondents communicated a pervasive sense of fatalism towards AIDS: “AIDS is not real. So long as God says it is your time—that is your time, even if you do not use a condom, if it is not your time, you will not die.”

### Recommendation for inclusion in HIV-prevention curriculum

After the discussion about sexual risk behaviours, focus-group participants provided suggestions about what should be included in an effective HIV-prevention curriculum for in-school youths.

One male youth commented:

I think the pressure from parents should be added. Some of our parents, especially after the war, can force their children to love “big people” so that they can bring things into the house.

One female commented:

… some of the things you really should talk about is parents forcing daughters to go on the street and look for money.

## DISCUSSION

The findings of the study indicate that the transactional nature of sexual encounters and the commoditization of female adolescent sexuality should be considered when developing HIV-prevention interventions. While transactional sex occurred among adolescents of similar age, emphasis was placed on sexual encounters with older, more financially-secure males to obtain cash, food, clothing, and school-fees. The age and economic asymmetries (42), material goods provided (13, 19), and the contradictory expectations and pressures from parents (12) are consistent with findings of other studies of transactional sex in sub-Saharan Africa. We found that the perception of the potential ‘benefits’ of transactional sex can influence a young woman's decision to become sexually-active. The promise of Western commodities and associated higher social status has been discussed in other studies of transactional sex (11, 13, 43) and were found to be strong motivators to engage in transactional sex in our study. Refusing sex or insisting on condom-use was reported to lead to possible sexual violence, consistent with findings of many studies in sub-Saharan Africa (17-19).

Our findings suggest that female adolescents in Liberia should not be viewed as simply victims of transactional sex. Similar to the findings of study by Wamoyi and colleagues in Tanzania, young girls are making choices, from the limited options they have, to engage in transactional sex to address a continuum of needs (26). They are described as choosing between age-concordant or discordant partners based on who can provide greater access to resources. Adolescent males are also willing participants in the transactional sex economy. They attested to male dominance over sexual encounters and felt in competition with older, more affluent males because they lacked access to the cash economy.

Similar to Luke and Katz's review of the transactional sex literature (44), a few focus-group participants described perceived risks from engaging in transactional sex. The risks communicated by the adolescent females included early pregnancy, lack of the opportunity to attend school if pregnant, sexual violence, and reduced prospects for marriage but these did not seem to outweigh the material benefits from transactional sex. Males did not perceive any risk for themselves when engaging in transactional sex.

In our focus groups, condom-use was viewed as a way to prevent early pregnancy. Condom-use was not motivated by a fear of HIV transmission. Adolescent females described discomfort with using condoms. They expressed fear that condoms would reduce fertility and, if insisted upon, could result in physical violence. Males indicated that they could dictate the terms of the sexual encounter and that condom-use would be viewed as accusing their partner of prostitution. These attitudes are reinforced by reports of low condom-use. According to the Liberia Health and Demographic Survey (2007), 12% of females and 15% of males aged 15-25 years use condoms (7).

The findings of our focus-group discussions indicate that video-clubs are potential high-risk settings frequented by youths and that transactional sex tends to occur in exchange for club entry. Sexual violence was a persistent concern of the female adolescents. Rape could occur if a female refused to have sex in a transactional encounter, and gang rape scenarios were described. In contrast, males viewed sexual violence as emerging from male desire, women's provocative clothing, and social circumstances. Sexual violence was pervasive during the two decades of civil conflict and is likely to have shaped adolescent attitudes. A study conducted by the World Health Organization in 2004 found that young girls during the civil conflict were taken by fighters as “bush wives”, cooks, cleaners, and sex slaves, and 75% of female respondents reported being raped (9). Although efforts led by international (30) and governmental actors are underway to reduce gender-based violence in Liberia, sexual violence remains a persistent component of Liberian culture (8). Results of ethnographic studies of sexual violence suggest that contracting economies in sub-Saharan Africa and lack of job opportunities may fuel male expression of power through sexual violence when other avenues to assert status are not available (25). This may certainly be the case in Liberia where GNP had fallen drastically as a result of the war (1). Young males—many coscripted as child soldiers during the war and having little access to schools—now find themselves with limited skills to enter the formal economy (45). Expression of male power through sex violence may diffuse to adolescents, further fuelling its acceptance.

The findings of our focus-group discussions indicate that the participants were disappointed by their parents and the broader elder community for condoning transactional sex and suggest that behavioural interventions should include parental pressures to engage in transactional sex. Past traditions of bride wealth, although no longer followed, prescribed that the groom's fami-ly pay the bride's family, often with cattle, in exchange for the bride with the implicit assumption that sex was highly valued and should not be given away free of charge. Such traditions may greatly influence permissive parental attitude towards transactional sex. In fact, it has been suggested that transactional sex may be viewed as a modern form of bridewealth where the payment is no longer between families but is negotiated between sex partners as sub-Saharan African countries shift from agricultural to consumer, cash-driven economies (46).

Based on the findings of focus-group discussions, we adapted the Making Proud Choices! curriculum to include role-plays to help young females articulate their future goals when confronted with familial pressure to engage in transactional sex and to help practise sexual refusal and condom-negotiation skills when faced with older males with greater sexual and financial power. Data-collection and analysis of the impact of this curriculum component on transactional risk behaviours are still underway.

### Implications for HIV prevention

The findings of the study suggest that we need to revise stereotypical notions of male dominance and female subordination in transactional sex encounters. These stereotypes disempower female adolescents and prevent the development of interventions that leverage female agency. School settings were perceived as protective for female adolescents allowing them to pursue their goals and escape pregnancy and early marriage. However, to stay in school often requires engaging in transactional sex to pay for books and uniforms. The President of Liberia instituted a policy in 2004 to waive tuition-costs for elementary school, made attendance compulsory, and revealed plans to cut secondary school-fees and reduce teacher-initiated sexual violence. Due to competing fiscal demands, many of these secondary school initiatives are yet to be implemented (47). An intervention, pilot-tested in Kenya, found that paying school-fees and providing other supports for orphans increased school attendance (48). Similar approaches could be adapted for Liberian adolescents to reduce the need to engage in transactional sex, breaking this cycle of dependency and risk.

In her review of the circumstances of sexual exchange in South Africa, LeClerc-Madlala suggests that only when women can participate in the economy will they be able to relinquish the financial rewards that are fundamental to their relationship with men (28). Structural interventions that train adolescent females in income-generation to cover school-related fees, household needs, or western commodities (49) should be explored for Liberian youths.

Further, we need to move beyond individual-centred interventions and gain a deeper understanding of how adolescent sexual behaviours are shaped by the larger sexual economy. Future research in Liberia is needed to examine the presence of multi-national NGOs, peacekeeping infrastructure and its entertainment economy and their impact on sexual economy of Liberia (10). Developers of HIV-prevention interventions need to be cognizant of the impact of these larger structural forces on shaping attitudes, norms, and behaviours of adolescents.

It has also been proposed that, in countries where transactional sex is deeply embedded, we should look to harm-reductive approaches within transactional sex encounters such as reducing the number and age disparities of transactional sex partners (26, 46). Based on the findings of the present study, we would also propose increasing condom-use self-efficacy and condom-related knowledge, explicitly separating condom-use from fears of infertility and building condom-negotiation skills in age and power-discordant relationships. We also need to consider adolescent males’ lack of perceived risk associated with transactional sex or unprotected sexual intercourse and to heighten their awareness of risks of HIV and other sexually transmitted infections.

Last, community-based interventions that are gender-transformative, like *Stepping Stones*, that have been implemented in sub-Saharan countries, can help build more gender and resource-equitable relationships to reduce transactional sex-related attitudes and behaviours (50). Such interventions could target parental attitudes, male perceptions of masculinity and, more generally, the family and community context. Such approaches should be considered when adapting HIV-prevention interventions to the highly-gendered realities young Liberians face.

### Limitations

These qualitative findings are based on a convenience sample of youths, aged 13-19 years, who attended one public school in Monrovia. One school was selected for the formative phase of the research, given the constraints of our study and to limit contamination to the schools in our randomized controlled trial. Approximately 59% of females and 66% of males are attending primary or secondary school in Liberia (51). However, given the limited research about the sexual risk behaviours of in-school and out-of-school adolescents, we cannot assume that these findings can be generalized to all Liberian adolescents in this age-group or to the larger Liberian school population.

## ACKNOWLEDGMENTS

The authors gratefully acknowledge funding support for this research from the National Institute of Mental Health (Grant No. R21 MH 82666-01) of the National Institutes of Health, Bethesda, MD, USA. They express their gratitude to the young people who participated in the study and the Liberian research team.
